# D-dimer as a predictive and prognostic marker among COVID-19 patients

**DOI:** 10.15537/smj.2022.43.7.20220213

**Published:** 2022-07

**Authors:** Ahmed M. E. Elkhalifa

**Affiliations:** *From the Department of Public Health, Health Sciences College, Saudi Electronic University, Riyadh, Kingdom of Saudi Arabia, and from the Department of Haematology, Medical Laboratory Sciences College, University of El Imam El Mahdi, Kosti, Sudan*.

**Keywords:** D-dimer, thrombocytopenia, platelets count, Najran

## Abstract

**Objectives:**

To examine D-dimer, coagulation profile, and platelet count among patients hospitalized with coronavirus disease-19 (COVID-19) and compare them to findings from non-COVID-19 subjects.

**Methods:**

The participants in this retrospective hospital-based observational study design included 112 confirmed diagnosed with COVID-19 who were admitted to King Khaled Hospital, Najran, Saudi Arabia, and another 112 non-COVID-19 subjects as a comparative group. Laboratory investigations, demographic and clinical records were obtained from participants’ electronic indexed medical records. Coronavirus disease-19 diagnosis was confirmed according to positive real time polymerase chain reaction assay carried out at the hospital’s central laboratory, where samples were extracted from a nasopharyngeal swab. Pneumonia related to COVID-19 is classified as critical, severe, moderate, mild, and asymptomatic whereas thrombocytopenia was marked when the platelet count was <150.00×10^9^/L. Suitable statistical analysis was applied to determine possible differences between the findings from the 2 groups.

**Results:**

The D-dimer and activated partial thromboplastin clotting time mean values were significantly elevated (*p*<0.001). The international normalized ratio and platelet count mean values confirmed a significant decrease (*p*<0.001). Thrombocytopenia was found 9 times in COVID-19 higher than in the non-COVID-19. D-dimer and prothrombin time mean values increased significantly among the COVID-19 patients with all patterns of symptoms on admission (*p*<0.001).

**Conclusion:**

D-dimer mean values increased significantly in deceased COVID-19 and in hospitalized intensive care unit (ICU) wards patients (*p*<0.001), indicating a potential predictive and prognostic severity marker, particularly among COVID-19 patients in the ICU.

**T**he novel Coronavirus disease-19 (COVID-19) aggressively spread throughout the world across various strains. Human-to-human transmission is obtained through contact with infected person droplets.^1^ Between the first recorded case in December 2019 and March 12, 2022, 455.7 million patients had been infected worldwide, with 6 million deaths reported.^2^ The first recorded case of COVID-19 disease in Saudi Arabia was registered on March 2, 2020, hence, thousands of cases were identified each day over the following months.^3-5^ After 2 years (by March 12, 2022), the number of infected patients had reached 748,624 cases, and there had been a total of 9017 deaths.^3^ Fortunately, due to comprehensive awareness programs, responsible decision-making, and applying effective and rigorous precautionary measures, a higher percentage of patients (97.6%) were cured and the mortality rate decreased to 1.2%, 9252 (1.2%) patients suffered mild COVID-19 symptoms, and only 313 (0.04%) patients were hospitalized with critical COVID-19.^3^ Furthermore, a successful comprehensive vaccination program using different types of vaccines was applied with 61.8 million doses given, including basic and booster doses distributed across all areas of Saudi Arabia.^3^

Coronavirus disease -19 patients usually present with respiratory compromise that may progress to primary complications including severe COVID-19 illness, cardiac disease, respiratory distress, organ failure, watery diarrhoea, and intravascular coagulopathy with even sudden death.^1,6-9^ Furthermore, abnormal coagulation parameters of COVID-19 patients were reported earlier in Chinese patients hospitalized in Wuhan, China. Approximately 36% were found with elevated D-dimer levels, 6% had higher activated partial thromboplastin time (aPTT) values, 12% had thrombocytopenia, and 5% had elevated prothrombin time (PT) values.^10^ Critically, COVID-19 disease patients were reported to have antiphospholipid antibodies, coagulopathy, and increased thrombosis.^11^

A single study on Saudi patients, examined coagulation parameters in cases with COVID-19 and control subjects, and it recorded a significant increase in D-dimer, aPTT, international normalized ratio (INR), and other coagulation factors.^12^ A similar study, confirmed a hyper coagulant profile among patients with severe COVID-19.^13^ Early recognition of these abnormal findings may be valuable for predicting the severity of the disease, supporting patients, and improving treatment and clinical outcomes.^14^

Prolonged coagulation factors may cause disseminated intravascular coagulation (DIC) as a marked increased risk for thromboembolic complications.^9,15^ Moreover, elevated PT and D-dimer may be allied with the highest mortality rates prognostic value.^9,16-19^ Platelet count is considered an available and simple biomarker; however, thrombocytopenia has been identified as a signiﬁcant indicator for mortality risk factors in severe patients.^20,21^ Therefore, the current study aims to analyze the coagulation parameters, D-dimer level, and platelet count among patients hospitalized with COVID-19 and compare them to findings from non-COVID-19 subjects. Furthermore, it sets out to analyze findings from COVID-19 patients according to their pattern of symptoms on admission and at their end-situation.

## Methods

An observational retrospective hospital record-based study design was carried out at King Khalid Hospital, Najran, Saudi Arabia, from September to December 2020, although it should be noted that the patients for the study were hospitalized during August 2020. The laboratory findings of the confirmed hospitalized patients included D-dimer, platelet count, PT, aPTT, and INR values, which were compared with non-COVID-19 subjects’ results. In addition, a comparison was carried out according to COVID-19 patients’ pattern of symptoms on admission and at their end-situation to examine whether it could be useful as prognostic and predictive severity markers.

All confirmed diagnosed COVID-19 patient’s files attended at King Khalid Hospital, Najran, Saudi Arabia, during August 2020 and had completed target laboratory investigation parameters in their files records and absence of comorbidities, other viral infection and chronic diseases were included in this study. Patients recorded with history of any disease that would have a significant influence on coagulation profile were excluded, as were those with history of cardiovascular disease, liver disease, alcoholism, heparin, aspirin, and warfarin long-term medication use, current drug effects on coagulation and platelet count, and incomplete target parameters from laboratory investigations. The exclusion criteria were applied to the COVID-19 patients’ group.

The sample size for this study considered and picked all the 112 confirmed COVID-19 disease patients files who attended at King Khaled hospital, Najran, Saudi Arabia, during August 2020 and had completed target laboratory investigation parameters and met inclusion and exclusion criteria. Another 112 files of patients without COVID-19 and with no blood disease and coagulation disorders were chosen randomly according to the inclusion and exclusion criteria and recruited as a comparative study subjects group.

Laboratory investigations, demographic and clinical records were obtained from participants’ electronic indexed medical records during the time that they were hospitalized (August 2020). Coronavirus -19 diagnosis was confirmed according to positive RT-PCR assay carried out at the hospital’s central laboratory by Roche/cobas 6800 System (Roche® Life Science Products). The samples were extracted from a nasopharyngeal swab according to World Health Organization (WHO) interim guidance.^22^

Pneumonia related to COVID-19 is classified as critical, severe, moderate, mild, and asymptomatic, whereas thrombocytopenia was marked when the platelet count was <150.00×10^9^/L.^23,24^

According to the principles of Helsinki Declaration, before the research process started, the research protocol was checked and approved by Saudi Electronic University Institutional Review Board committee (No.: SEUREC-CHS20113).

### Statistical analysis

The extracted data coded, revised, and entered using the Statistical Package for the Social Sciences, version 26.0 (IBM Corp., Armonk, NY, USA). Variables were expressed according to the test of normality results. T-test was carried out for comparing the 2 study groups’ findings. Fisher exact test was carried out due to small numbers as a result of sample fragmentation on multiple categories to compare the COVID-19 patients’ pattern of symptoms on admission and at their end-situation. Binary logistic regression was applied to evaluate D-dimer, platelet count, and coagulation parameters’ role in predicting severity values of COVID-19 patients. Receiver operating characteristics (ROC) curves were carried out by MedCalc, version 19.0.4 (bv, Ostend, Belgium) to explain the role of D-dimer as a predictive marker among COVID-19 patients. Statistical values less than 0.05 deemed significant.

## Results

Table1 indicates that the age range of the study group and comparative group ranged between 25-90 years old (51.66±15.3) for COVID-19 patients and 54.96±18.6 years for non-COVID-19 patients. Males represented the majority of the study participants (86.8%). Two-thirds (66.1%) COVID-19 patients were between 30-60 years old. Most of the participants were Saudi, and the remaining patients were from other nationalities working as expatriates in Saudi Arabia. A total of 67 (59.1%) of the COVID-19 participants entered the hospital with moderate, critical, or severe symptoms, while the remaining 45 (40.1%) attended to the hospital with asymptomatic or mild symptoms. Three-quarters (75%) of the COVID-19 patients recovered, 17 (15.2%) remained hospitalized and isolated in intensive care unit (ICU) wards, and 9 (8%) patients unfortunately deceased.

**Table 1:** - Coronavirus disease-19 (COVID-19) and comparative subjects’ demographic data, symptoms, and end situation outcomes.

Variables	COVID-19	Comparative subjects
*Gender*		
Female	26 (23.2)	47 (41.9)
Male	86 (76.8)	65 (58.1)
***Age (years)***		
<30	5 (4.5)	18 (16.1)
30-60	74 (66.1)	45 (40.2)
>60	33 (29.5)	49 (43.8)
Mean±SD	51.66±15.3	54.96±18.6
Range	25-90	25-90
***Nationality***		
Saudi	66 (58.9)	73 (65.2)
Egyptian	5 (4.5)	3 (2.7)
Yemeni	13 (11.6)	10 (8.9)
Pakistani and Filipina	8 (7.1)	9 (8)
Bangladeshi	6 (5.4)	1 (0.9)
Indian	4 (3.6)	2 (1.8)
others	10 (8.9)	14 (12.5)
***Symptoms***		
Mild and asymptomatic	45 (40.1)	NA
Moderate, severe, and critical	67 (59.9)	NA
***Outcomes situation***		
Recovered	86 (76.8)	NA
ICU patient	17 (15.2)	NA
Deceased	9 (8.0)	NA

Values are presented as a number and precentage (%). NA: not available, SD: standard deviation, ICU: intensive care unit

Table 2 shows a significant higher aPTT and D-dimer mean values in COVID-19 group versus the non-COVID-19 (*p*<0.001). By contrast, platelet count and INR mean values confirmed a significant decrease in COVID-19 (*p*<0.001). Thrombocytopenia was elevated 9 times among COVID-19 than in non-COVID-19 participants (*p*<0.005). Prothrombin time mean values showed non-significant (*p*<0.620).

**Table 2:** - Coronavirus disease-19 and comparative subjects’ coagulation parameters and platelets count results on admission.

Parameters	COVID-19	Comparative subjects	T-test	*P*-values
PT (seconds)	13.15±1.46	13.30±2.70	0.497	0.620
APTT (seconds)	33.69±5.42	30.63±7.47	3.47	<0.001^*^
PLTs count (x10^3^)	243.38±103.68	287.72±89.07	3.43	<0.001^*^
D-dimer (μg/mL)	1.05±0.71	0.16±0.06	13.70	<0.001^*^
INR	1.08±0.13	1.15±0.23	2.82	0.005^*^

Values are presented as mean ± standard deviation (SD).

* Significant, PT: prothrombin time, APTT: activated partial thromboplastin clotting time, PLTs: mean platelets, INR: international normalized ratio

Table 3 shows the gender and age factors of the COVID-19 patients. The age factor was found to be highly significant to the tune of 9.85 times higher among older patients (*p*<0.006).

**Table 3:** - Gender and age factors of the coronavirus disease -19 patient’s outcomes situation at the end of the study period (N=112).

Gender and age factors	Outcomes situation		χ^2^	*P*-values
	Recovered	Non-recovered		
*Gender*				
Female	24	2	1.05	0.514
Male	73	13		
***Age***				
>60 years old	23	10	9.85	<0.006^*^
≤60 years old	74	5		

Values are presented as numbers. ^*^Significant (Fisher exact test)

Table 4 shows a significant increase in D-dimer and PT mean values among moderate, severe, and critical hospitalized patients. D-dimer mean values indicate a significant increase among ICU and deceased versus recovery and discharged patients (*p*<0.001).

**Table 4:** - Coronavirus disease-19 patient’s pattern of symptoms on admission and the outcomes of the patient’s situation concerning coagulation parameters and platelet count.

Parameters	PT (seconds)	aPTT (seconds)	PLTs count (x10^3^)	D-dimer (μg/mL)	INR
Moderate, severe, and critical symptoms	13.44±1.5	33.82±5.96	243.02±109.89	1.29±0.82	1.09±0.14
Asymptomatic and mild symptoms	12.72±1.29	33.50±4.56	243.91±94.9	0.75±0.47	1.05±0.10
T-test (*p*-value)	3.19 (0.008)^*^	0.321 (0.749)	0.044 (0.956)	4.293 (<0.001)^*^	0.208 (0.110)
ICU and deceased patients	13.54±2.03	34.22±6.58	219.78±108.00	1.47±0.75	1.10±0.19
Recovery patients	13.02±1.20	33.51±5.01	251.25±101.94	0.95±0.72	1.07±0.10
T-test (*p*-value)	1.63 (0.104)	0.601 (0.549)	1.39 (0.165)	3.32 (<0.001)^*^	1.07 (0.283)

Values are presented as mean ± standard deviation (SD).

* Significant, PT: prothrombin time, aPTT: activated partial thromboplastin clotting time, PLTs: mean platelets, INR: international normalized ratio, ICU: intensive care unit

Table 5 indicates that binary logistic regression analysis confirmed the role of D-dimer as an obvious significant prognostic severity factor among COVID-19 (*p*<0.001).

**Table 5:** - Coagulation parameters and platelets count on admission assessment using binary logistic regression analysis

Coagulation parameters and platelets count	B	S.E.	Exp(B)	*P*-value
D-dimer (μg/mL)	0.020	0.004	1.021	<0.001^^*^^
PLTs count (x10^3^)	-0.006	0.005	.994	0.289
PT (seconds)	-0.145	0.313	.865	0.643
INR	-2.112	2.805	.121	0.451
APTT (seconds)	0.077	0.083	1.080	0.356
Constant	-2.791	4.117	.061	0.498

* Significant, PT: prothrombin time, APTT: activated partial thromboplastin clotting time, PLTs: mean platelets, INR: international normalized ratio

**Figure 1:**
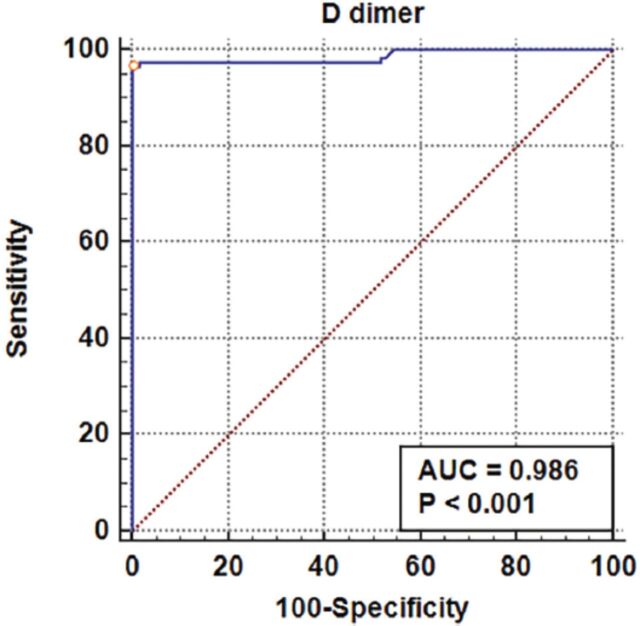
- Receiver operating characteristic curve analysis of coronavirus disease-19 patients D-dimer. AUC: area under the curve

In Figure 1, the ROC curve determines D-dimer as a predictive marker in COVID-19 (area under curve [AUC]=0.986, specificity=100%, sensitivity=96.43%, cut-off value of >2.55 μg/Ml, Youden index=0.9643, and *p*<0.001).

## Discussion

The novel COVID-19 disease and its rapidly spreading mutations are characterized by a wide severity range from asymptomatic to critical and even death.^25^ Monitoring coagulopathy and early detection of coagulation laboratory biomarker abnormalities is essential for the prediction and prognosis of disease severity among hospitalized patients and could help the improvement of clinical outcomes of COVID-19 patients.^9,15,25^ Elevated D-dimer and abnormal PT and aPTT have been used as coagulating factors for the early detection of DIC and indicated a hyper coagulation and higher risk of thrombosis.^26^ Furthermore, thrombocytopenia is associated with thromboembolic complication risk reported among severe COVID-19.^27^ The infected patients’ mean age was 51.66 years old, which was consistent with mean ages mentioned in previous studies reported that COVID-19 infection dominated among the older population and was lower among the young population.^27-29^ The age factor was found to be highly significant, to the tune of 9.85 times higher among older patients (*p*<0.006). However, older age was recorded as one of the features associated with increased chance of death.^30^ This study confirmed that 76.8% of patients were males and 23.2% were females consistent with previous studies that observed male predominance and higher susceptibility among those with older age. Females’ low susceptibility to COVID-19 infection may be attributed to their stronger immune response.^10,14,27-29,31^ Approximately 59.9% of the COVID-19 patients group attended hospital with moderate, severe, or critical symptoms, which may be related to their weak immune response and older age, as reported in previous studies.^28,29,32,33^ A total of 45 (40.1%) COVID-19 patients were admitted with asymptomatic or mild symptoms, which may be attributed to their good immune response, as was consistent with some reports that recorded a higher rate of asymptomatic and mild symptoms among patients at hospital admission.^34^ In addition, the most observed symptoms were cough, fever, shortage of breath, and taste and smell disorders, which was in line with the findings of other studies.^10^ D-dimer mean values were found to be elevated versus those of non-COVID-19 patients, with a higher significance among deceased and ICU patients. D-dimer mean values among the moderate, severe, and critical COVID-19 patients confirmed significant differences compared to mild symptoms and asymptomatic patients (*p*<0.001), which was consistent with previous research in which the disease severity was considered.^9,16^ Researchers have confirmed elevated D-dimer to be one of the laboratory markers important for patients hospitalized with COVID-19, with a potential association with higher risk incidence of thrombotic complications concerning ICU and even death among COVID-19 patients with D-dimer levels exceeding 1μg/ml at hospital admission.^18-20,26,30,35^ Activated partial thromboplastin clotting time showed a significant increased (*p*<0.001), which was consistent with previous research findings but in contrast with another study that reported a non-significant difference in aPTT.^26,36^ Platelet count confirmed a significant decrease (*p*<0.001), and thrombocytopenia was 9 times greater in COVID-19 than non-COVID-19 subjects (16.1% versus 1.8%). These findings were in agreement with large-scale and other retrospective studies that have reported thrombocytopenia in 20% of COVID-19 patients and indicted thrombocytopenia in scoring systems for severity of the disease.^21,28,29,34^ Furthermore, a higher risk of death and bleeding was reported among patients with hypoxemia and a progressive decrease in platelet count.^9,21^ The PT results showed a non-significant difference, which was consistent with a previous study that reported a normal PT values.^1^ The INR mean values indicated a significant decrease in COVID-19 (*p*<0.001), in contrast with previous research that reported a mild prolongation of INR values among COVID-19 patients.^14^ Prothrombin time and D-dimer mean values increased significantly in the COVID-19 moderate, severe, and critical symptoms compared with asymptomatic and mild symptoms (*p*<0.001), in line with published studies that mentioned increased levels of PT and D-dimer associated with COVID-19 disease progression and higher mortality.^9,17^ Further statistical analysis was carried out to assess the effect of D-dimer, PT, aPTT, INR, and platelet count for the 2 groups using binary logistic regression analysis. Activated partial thromboplastin clotting time, PT, platelet count, and INR items were removed from the equation in the first step, and D-dimer remained the only parameter in the equation (*p*<0.001) that signaled a potential prognostic and severity marker role among COVID-19 patients, and ROC confirmed D-dimer to have highest AUC of 0.986. These predictive and prognostic severity marker roles of D-dimer were consistent with recently published studies that suggested a contemporaneous presence regarding high D-dimer levels, which are considered a potentially important severity predictor marker for COVID-19 disease associated with death, mainly among mechanical ventilation patients.^12,13,31,37^

### Study limitations

A number of coagulation parameters, including factor V111, fibrinogen, thrombin time, and Von Willebrand factor, were not included due to their absence in the routine laboratory investigation for our COVID-19 patients’ study group, so it is highly recommended for further studies to include all the coagulation-related parameters.

In conclusion, D-dimer mean values, thrombocytopenia, and prolonged coagulation factors were significantly affected by COVID-19 disease, particularly in critical and severe cases associated with a poor prognosis. Early recognition of abnormal coagulation findings and monitoring of coagulopathy is essential and highly recommended to support COVID-19 patients, improve their clinical outcomes, and reduce severe complications.
